# Camera recognition with deep learning

**DOI:** 10.1080/20961790.2018.1485198

**Published:** 2018-10-17

**Authors:** Eleni Athanasiadou, Zeno Geradts, Erwin Van Eijk

**Affiliations:** a Department of Forensic Science University of Amsterdam, Amsterdam, The Netherlands;; b Netherlands Forensic Institute Den Haag, Den Haag, The Netherlands

**Keywords:** Forensic sciences, camera identification, clustering, individualization deep learning

## Abstract

In this paper, camera recognition with the use of deep learning technique is introduced. To identify the various cameras, their characteristic photo-response non-uniformity (PRNU) noise pattern was extracted. In forensic science, it is important, especially for child pornography cases, to link a photo or a set of photos to a specific camera. Deep learning is a sub-field of machine learning which trains the computer as a human brain to recognize similarities and differences by scanning it, in order to identify an object. The innovation of this research is the use of PRNU noise patterns and a deep learning technique in order to achieve camera identification. In this paper, AlexNet was modified producing an improved training procedure with high maximum accuracy of 80%–90%. DIGITS showed to have identified correctly six cameras out of 10 with a success rate higher than 75% in the database. However, many of the cameras were falsely identified indicating a fault occurring during the procedure. A possible explanation for this is that the PRNU signal is based on the quality of the sensor and the artefacts introduced during the production process of the camera. Some manufacturers may use the same or similar imaging sensors, which could result in similar PRNU noise patterns. In an attempt to form a database which contained different cameras of the same model as different categories, the accuracy rate was low. This provided further proof of the limitations of this technique, since PRNU is stochastic in nature and should be able to distinguish between different cameras from the same brand. Therefore, this study showed that current convolutional neural networks (CNNs) cannot achieve individualization with PRNU patterns. Nevertheless, the paper provided material for further research.

## Introduction

Nowadays, because of the ambiguity of digital cameras, mobile cameras, video cameras and so on, the number of images is huge and these images are really important in crime investigation. In digital forensics, it is important to identify the source of an image in order to link it with a specific digital camera or link images with a common source. It is also important to prove the authenticity of an image, as there is a possibility to be computer-generated. Like in ballistics the forensic expert matches a bullet to a specific gun, in the same manner, an image can be related to a specific type of camera.

Each different camera has different noise patterns, the so-called photo-response non-uniformity (PRNU) noise patterns. These are essential parts of all digital imaging sensors because of slight variations between pixels. The PRNU is considered as the fingerprint of the camera. Each camera has a device that converts the light into electrical signal which is the imaging sensor. Charged coupled device (CCD), complementary metal-oxide-semiconductor (CMOS), junction field effect transistor (JFET) and Foveon X3 are some of the popular imaging sensors with the CCD being the most frequent in use. To process the picture, the sensor is divided in pixels. All imaging sensors, exhibit unique PRNU patterns because of the slight variations between individual pixels and their ability to convert the light (photons) into electrical signal (electrons) [1]. This fingerprint is present independently of the camera optics, camera settings or scene content with the exception of completely dark images. As a result, even if someone changes the camera settings, both images (before and after the change) will exhibit the same PRNU pattern [2]. Thus, from a questioned image, the PRNU noise pattern can be extracted and checked against a reference image. It is important to mention that two digital cameras from the same brand have different PRNU as these patterns are stochastic in nature and unique to each sensor.

A deep learning approach can be implemented to train a system that automatically recognizes PRNU patterns in order to identify the source camera. In deep learning approaches, there are different layers that are composed of neurons communicating with each other. As a result, the “previous” layer is training the next one with the acquired information [3].

The aim of this research project is to perform and evaluate camera identification with the use of PRNU noise pattern and deep learning techniques. During this study, it was examined whether this combination could provide promising results using cropped images.

## Forensic relevance

In forensic science, camera identification mainly contributes to child pornography or terrorism cases. The first step of the investigation is to prove that a certain image has been captured using a real camera rather than being computer-generated [1]. After that, identification of the camera is attempted by comparing a suspect's camera with a reference. In another case where no camera has been seized from a suspect, the extracted PRNU pattern can be compared with an existing database of previously collected PRNU patterns in order to examine whether or not there were other images from other cases with the same camera. As child pornography cases have a large amount of images from unknown sources, if there is a suspect and a suspect camera available, it is important to determine if any of the images originated from the suspect's camera. In addition, the quality of the image should be sufficient, while the computation time should be preferably low.

Other possible cases to which camera identification can contribute are extortion or bullying. In both occasions, a video or a photo may be sent to a victim for a financial or a psychological attack. Even if the image is processed, the identification is still possible [4]. However, if the file is uploaded at a video sharing website, like YouTube, the PRNU noise pattern will be degraded due to the extra compression process. Consequently, it will be more difficult to trace and identify the source [5]. The camera identification can also contribute to homicide cases where the perpetrator has immortalized the victims or his/her actions.

## Current methods

There are several ways to identify the camera. One of the simplest methods is to examine the electronic file itself. From headers, for example EXIF headers or the quantization table in JPEG headers, it is often possible to retrieve important information about the type of the camera or under which conditions the image was taken. The limitation of this approach is that even though there are cameras that are claimed to be “secure” and have added a visible or invisible watermark with time stamps, it cannot be used for all the cameras. Also, the header data, due to manipulation or re-saving of the image in a different format, may not be correct or even available. As a result, there is lack of credibility of these traces.

Another approach is the investigation and comparison of defected pixels. These are hot or dead pixels that can link images or identify the camera. However, some camera models do not contain defected pixels or fix this “flaw” by eliminating these particular pixels in a post-process of the image. Another problem of this approach is that some defective pixels may not be visible due to the scene that is shot. Thus, the camera itself is needed and may not be available [6].

An approach that uses PRNU noise patterns was introduced by Lukas et al. [4] in 2006. In this paper, the process of extracting the PRNU noise was described. The extracted noise was compared with a reference camera if it was available, or with PRNU noise from an existing database. The authors stated that the proposed method can be used for reliable camera identification; however, operations like resizing or rotating the image may increase the computation complexity [4]. Based on this method, a new tool was created for the extraction and the comparison of the PRNU noise pattern by the Netherlands Forensic Institute, “PRNU Compare” [7,8].

A combination of camera identification and deep learning approach was published on 2016 by Tuama et al. [3]. The researchers used “the artefacts that exist in the camera pipeline to collect specific features manually and then use them to distinguish between camera models or individual devices”. The research increased the efficiency and the authors claimed that the more layers a deep learning application has, the more promising the results will be.

## Deep learning

Deep learning is a sub-field of machine learning which uses layers of artificial neural networks in order to train a system, identify and classify items. In this case, it was used to process the PRNU noise patterns obtained from the cameras and the images to be able to distinguish and identify them. As it was mentioned in the review paper of Lecun et al. [9], a proper definition is that “Deep learning allows computational models that are composed of multiple processing layers to learn representations of data with multiple levels of abstraction”. Basically, the system is trained like the human brain to identify the similarities and differences between objects.

**Figure 1.**
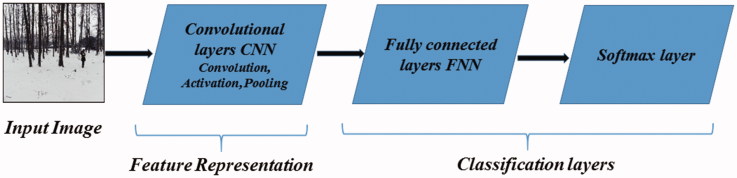
General structure of convolutional neural network [3].

Deep learning techniques are used to identify objects in images, recognize images but also classify them. It is also able to identify faces successfully which is valuable for forensic science. For this research, deep learning with the use of convolutional neural networks (CNNs) was used. CNNs have many layers which consist of neurons. Each layer receives the output of the previous layer as an input, except the first one which is given an input. A general structure of CNNs is shown in Figure 1. Every CNN has four main operations.

### The convolution step

The primary purpose of this step is to extract features from the input image. Any image is able to be represented as a matrix of thousands of pixels. To extract the features from the input image another smaller matrix, also called filter or kernel which acts like feature identifier, slides over the input image in order to create a new matrix as output which records the position of a feature that is detected and called “Activation Map” or “Feature Map”. Before this stage is performed, it is important to decide two parameters. The first is the depth which is the number of kernels that are used by the convolutional layer and the second is the stride which is the number of pixels by which this feature detector is slide over the input matrix.

### Rectified Linear Units (ReLU)

The purpose of this step is to introduce non-linearity to a system which in the convolutional step computes linear operations. This is a crucial step since most of the real-world data are non-linear. As a result, ReLU, which is an activation function, is needed to convert them from linear operations to non-linear.

### The Pooling step

The main purpose of this step is to make the “Activation map” more adaptable by making it smaller. It actually reduces the dimensionality of each Activation map. However, the most important information is preserved. In other words, the amount of parameters is reduced. Pooling has three different options: max, average and sum [9].

### Fully connected layers

**Figure 2.**
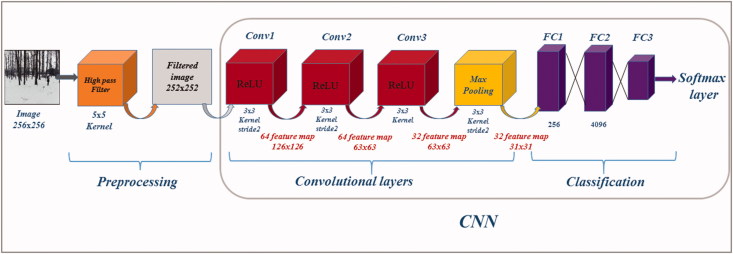
Layout of a convolutional neural network (CNN) model as proposed by Tuama et al. [3] illustrating the different layers/steps.

These three steps are the basic operations of any CNN. The last pooling layer output becomes the input to the next step which is the fully connected layer. The layer identifies to the given input, the features which are more correlated to a class. In the output layer a softmax activation function is used in the output layer which guarantees that the sum of the output probabilities on all the neurons of the layer will be one. This operation will convert the output to probabilities and is used at the end of the network [10]. Figure 2 shows a layout of a CNN model with its parameters.

## Materials and methods

A single graphics processing unit (GPU) card of type GeForce GTX 1080Ti manufactured by NVIDIA and NVIDIA DIGITS (https://developer.nvidia.com/digits), an Interactive Deep Learning GPU Training System, were used for the implementation of the project. DIGITS applies deep learning in an uploaded database and then classifies it. On the next step, the appropriate network is chosen depending on the size of the dataset. During the second step, the model is trained in order to extract features and find similarities and differences between the categories. In DIGITS, there are three given networks LeNet [11], AlexNet [12] and GoogleNet [13]. A graph is then provided informing the user of the success of the training. The parameters provided by the graph are the accuracy of the trained model, the loss of data during the process of training and validation. On the last step, the user uploads a single image and the program provides the “Top-5 predictions”.

**Figure 3.**
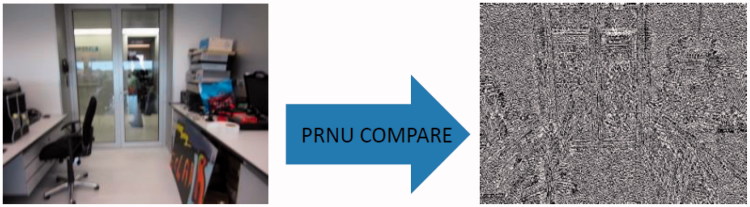
Example of photo-response non-uniformity (PRNU) pattern noise of Canon Ixus.

For the compilation of the database, an efficient and approved tool created by the Netherlands Forensic Institute (NFI), “PRNU Compare”, was used. This tool extracts and compares the PRNU noise patterns. Additionally, it has been implemented in MATLAB and works on the Java platform. With the use of “PRNU Compare” a database with PRNU noise patterns was created. An image of extracted PRNU is shown in Figure 3.

### Database collection

In order to start the camera identification with deep learning, a database is necessary with the appropriate material. In this case a data set of extracted PRNU noise patterns was used. A database of 27 different camera models, each of them containing multiple cameras (from the same model) was created. In total, there were 60 477 images. Several different data sets (subdivisions of the database) were then created for each different experiment. Each camera/category contained both natural and flat field images. In general, the best pattern is obtained by images from a homogeneously coloured and evenly illuminated surface, such as a white wall. These images are called flat field images [4]. Although flat field images give the most optimal results, natural photos can also be used to obtain a PRNU pattern.

### Selection of a pre-trained CNN

Camera identification with deep learning in cropped images was performed and evaluated. The input image was necessary to be as big as possible in dimensions because of the feature that have to be extracted from it. Furthermore, the used database contained 200 images per class which was also important for the selection of the CNN model.

For the following experiments, the AlexNet was chosen. Its basic advantage over LeNet is that AlexNet takes input images of dimensions 256 × 256 while LeNet takes 32 × 32 which is extremely small for the amount of information needed for the training. GoogleNet is too complicated to be implemented and modified as it contains 22 convolutional layers while AlexNet contains 5. It is presented as a model for a very deep training procedure which is better to be used in classes containing more than 20 000 items. All the default networks give unsatisfactory results. As a consequence, the following modifications are crucial.

### Modifications to the selected CNN

In combination to the default network and the one suggested in [3], modifications were made to AlexNet. On three out of five convolution layers (Conv1–Conv3), the kernels and their size were changed. Table 1 shows the differences between the default AlexNet and the modified one. On Conv1 there were 96 kernels of size 11 × 11 which changed to 64 kernels of size 3 × 3 in order to produce 126 × 126 of size feature maps. On Conv2 only, the size of the kernels was changed from 5 × 5 to 3 × 3 and, on Conv3, there were 384 kernels which were reduced to 32 kernels of size 3 × 3. However, the other two convolutions and the fully connected layers were kept intact. The motivation to make these changes was the fact that the PRNU noise patterns contain a lot of details. The reduction of the number of kernels and their size helps to retain a lot of original pixel information.

**Table 1:** . Default AlexNet and modified AlexNet.

Convolution layers	Default AlexNet	Modified AlexNet
Conv 1		
No. of Kernels	96	64
Kernel size	11 × 11	3 × 3
Conv 2		
No. of Kernels	32	32
Kernel size	5 × 5	3 × 3
Conv 3		
No. of Kernels	384	32
Kernel size	3 × 3	3 × 3

## Experiment and evaluation

For the implementation of the experiments, 17 cameras were used from the NFI database. As it was mentioned earlier, a pre-processing of the photos was made so as to extract the PRNU noise pattern by using “PRNU Compare”. The list is given in Table 2. Since the purpose of DIGITS is to classify objects, in this research project, it was used to create classes of different cameras and subsequently identify them. Each class contained images of only one camera in order to attempt individualization.

**Table 2.** Cameras used in the experiments.

Camera	Original resolution	No. of images
Blackberry Curve 9300	1 600 × 1 200	200
Creative Live! Cam Video IM	640 × 480	200
Canon Ixus 220HS	640 × 480	200
Motorola V360	640 × 480	200
Panasonic Lumix SZ5	640 × 480	200
Philips SPC 200NC	320 × 240	200
Canon Powershot SX210	640 × 480	200
Sigma DP1S	1 872 × 1 248	200
Sweex WC001 100k USB	320 × 240	200
Vodafone 710	640 × 480	200
Blackberry Curve 9360	2 592 × 1 944	200
Logitech QuickCam Communicate STX	640 × 480	200
Nikon Coolpix L27	4 608 × 3 456	200
Samsung Galaxy S3 mini	2 560 × 1 920	200
Samsung ST30	1 024 × 768	200
Trust WB-1400T	320 × 240	200
Sony Cyber-shot DSC-S800	3 264 × 2 448	200
Total		3 400

### DIGITS' parameters

The images were cropped to 256 × 256 with the use of DIGITS in order to fit the network's conditions. The dataset was separated to three sub-sets manually: Training, Validation and Test set. From the database 75% was the Training set which was the main set used for the training procedure. The Validation set was the 10% of the database and it was used in order to test the created model. The rest 15% was the Test set which contained images that were not part of the training procedure. In general the Test set is used as real-world data in order to test the created model to unseen data. Furthermore, the models were trained with 0.002 5 base learning rate which affects the learning of the network's speed and 500 training epochs which is the number of passes through the training data.

## Results

### Experiment 1

**Figure 4.**
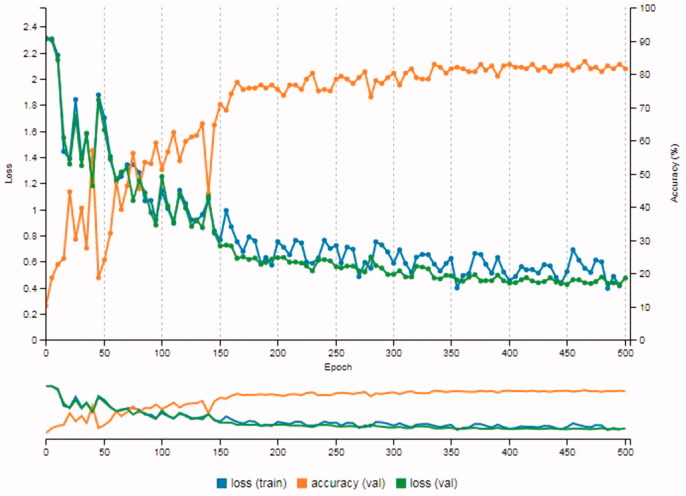
Results of Experiment 1.

The first 10 cameras given in Table 2 were used for the Experiment 1. As it was mentioned earlier, modifications were made to AlexNet as it was proposed by Tuama et al. [3]. Figure 4 shows the results from the Experiment 1. The accuracy value was 81%. Also, the loss (train), which is the error on the training set of data, was 0.41 and the loss (val), which is the error after running the validation set through the trained model, was 0.47.

The Test set, which contains images that are not a part of the training procedure, was used to identify the source camera of each image in the set, in order to provide the identification accuracy. For each category, there were 30 images. In DIGITS, the results were arranged in a confusion matrix shown in Table 3. From Table 3, we can see that the best identification accuracy was recorded in “Sigma DP1S” and “Sweex WC001 100k USB” which achieved 96.67% and “Creative Live! Cam Video IM” and “Philips SPC 200NC” with 93.33%. On the other hand, the lowest accuracy rate, 0%, was recorded in “Canon Ixus 220HS” and “Vodafone 710”. Furthermore, DIGITS provided a summary with “Top-1 accuracy (the percentage of the classifications that reached the ground truth)” and “Top-5 accuracy (the percentage of classification which did not reach the ground truth but the top 5 predictions)” which were 57.67% and 98%, respectively.

**Table 3.** Confusion matrix showing the number of photos identified for each category.

No.	Camera	1	2	3	4	5	6	7	8	9	10	Per-class accuracy (%)
1	Blackberry Curve 9300	23	0	0	0	0	0	0	7	0	0	76.67
2	Creative Live! Cam Video IM	0	28	0	1	1	0	0	0	0	0	93.33
3	Canon Ixus 220HS	0	0	0	0	26	0	1	3	0	0	0
4	Motorola V360	0	0	0	10	17	0	0	3	0	0	33.33
5	Panasonic Lumix SZ5	0	0	0	0	25	0	0	5	0	0	83.33
6	Philips SPC 200NC	0	2	0	0	0	28	0	0	0	0	93.33
7	Canon Powershot SX210	0	0	1	0	0	28	0	1	0	11	3.33
8	Sigma DP1S	1	0	0	0	0	0	0	29	0	0	96.67
9	Sweex WC001 100k USB	0	1	0	0	0	0	0	0	29	0	96.67
10	Vodafone 710	0	0	1	1	25	0	3	0	0	0	0

### Experiment 2

**Figure 5.**
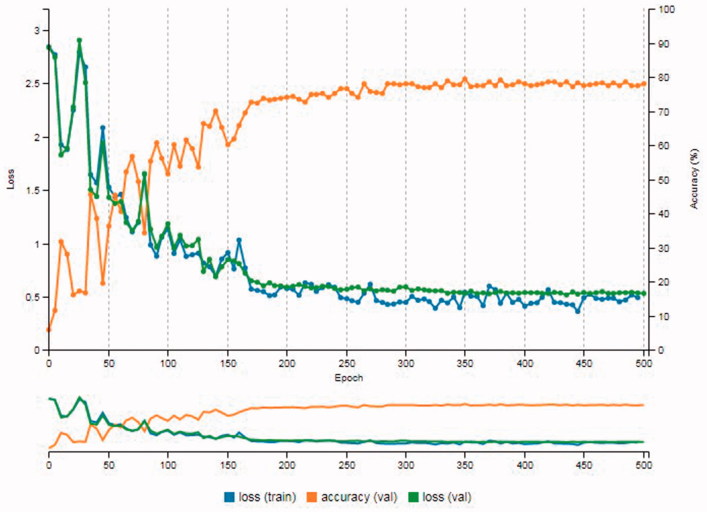
Results of Experiment 2.

The modified AlexNet was performed again with all 17 cameras given in Table 2. Figure 5 shows the results from the Experiment 2. With this, the accuracy value decreased to 78%. Also, both loss (train) and loss (val) increased to 0.55. As it was expected, the accuracy decreased as the number of models increased. In general, in machine learning, this is normal especially when the number of classes/categories increase. The fact that both loss (train) and loss (val) increased was also expected since the training process had more different classes to handle.

The confusion matrix, which is shown in Table 4, was also changed. The best identification accuracy was recorded in “Philips SPC 200NC”, “Nikon Coolpix L27” and “Trust WB-1400T” which achieved 100%. While the lowest accuracy rate which was 0%, was recorded in 11 out of 17 classes. From those 11 classes, six had been identified with almost 100% accuracy as “Samsung ST30” and 2 as “Nikon Coolpix L27”. For this reason, the next, Experiment 3, was performed to check the differences. Furthermore, DIGITS provided a summary with Top-1 accuracy and Top-5 accuracy which were 21.57% and 69.61%, respectively.

**Table 4.** Confusion matrix showing the number of photos identified for each category.

No.	Camera	1	2	3	4	5	6	7	8	9	10	11	12	13	14	15	16	17	Per-class accuracy (%)
1	Blackberry Curve 9300	0	0	0	0	0	0	0	0	0	0	0	0	0	30	0	0	0	0
2	Creative Live! Cam Video IM	0	17	0	0	0	0	0	0	0	0	0	0	0	0	13	0	0	56.67
3	Canon Ixus 220HS	0	0	0	0	0	0	0	0	0	0	0	0	0	0	30	0	0	0
4	Motorola V360	0	0	0	1	0	0	0	0	0	0	0	0	0	0	29	0	0	3.33
5	Panasonic Lumix SZ5	0	0	0	0	0	0	0	0	0	0	0	0	0	0	29	0	1	0
6	Philips SPC 200NC	0	0	0	0	0	30	0	0	0	0	0	0	0	0	0	0	0	100.00
7	Canon Powershot SX210	0	0	0	0	0	0	0	0	0	0	0	0	0	0	30	0	0	0
8	Sigma DP1S	0	0	0	0	0	14	0	2	0	0	1	0	0	0	0	0	13	6.67
9	Sweex WC001 100k USB	0	0	0	0	0	4	0	0	0	0	0	0	0	0	0	26	0	0
10	Vodafone 710	0	0	0	0	0	0	0	0	0	0	0	0	0	0	30	0	0	0
11	Blackberry Curve 9360	0	0	0	0	0	0	0	0	0	0	0	0	21	0	0	0	9	0
12	Logitech QuickCam Communicate STX	0	0	0	0	0	0	0	0	0	0	0	0	0	0	30	0	0	0
13	Nikon Coolpix L27	0	0	0	0	0	0	0	0	0	0	0	0	30	0	0	0	0	100.00
14	Samsung Galaxy S3 mini	0	0	0	0	0	0	0	0	0	0	0	0	27	0	0	0	3	0
15	Samsung ST30	0	0	0	0	0	0	0	30	0	0	0	0	0	0	0	0	0	0
16	Trust WB-1400T	0	0	0	0	0	0	0	0	0	0	0	0	0	0	0	30	0	100.00
17	Sony Cyber-shot DSC-S800	0	0	0	0	0	0	0	0	0	0	0	0	30	0	0	0	0	0

### Experiment 3

For this experiment, 15 out of 17 cameras were used leaving out “Samsung ST30” and “Nikon Coolpix L27” in order to test the hypothesis that these cameras are confusing the training of the CNN. The accuracy value, the loss (train) and loss (val) were similar to the Experiment 1. As a result, they were better than Experiment 2. Additionally, the Top-1 Accuracy and Top-5 Accuracy increased to 31.11% and 74%. In Table 5, it is presented the confusion matrix and, in Table 6, it is presented the comparison of the two experiments. Most of the percentages were increased with the exception of “Trust WB-1400T” which decreased from 100% to 83.33%. Even though, they have not increased significantly, it showed that indeed “Samsung ST30” and “Nikon Coolpix L27” were confusing the training procedure. This is probably because of common imperfections of the sensor which are included in the PRNU patterns and may be similar to many camera models. As a result, in Experiment 2, there were false identifications.

**Table 5.** Confusion Matrix showing the number of photos identified for each category.

No.	Camera	1	2	3	4	5	6	7	8	9	10	11	12	13	14	15	Per-class accuracy (%)
1	Blackberry Curve 9300	0	0	0	0	0	0	0	0	0	0	0	14	0	0	16	0
2	Creative Live! Cam Video IM	0	29	0	0	0	0	0	1	0	0	0	0	0	0	0	96.67
3	Canon Ixus 220HS	0	0	1	2	8	0	0	19	0	0	0	0	0	0	0	3.33
4	Motorola V360	0	0	2	12	0	0	0	16	0	0	0	0	0	0	0	40.00
5	Panasonic Lumix SZ5	6	1	0	0	15	0	0	7	0	0	0	0	0	0	1	50.00
6	Philips SPC 200NC	0	0	0	0	0	30	0	0	0	0	0	0	0	0	0	100.00
7	Canon Powershot SX210	0	0	2	3	16	0	9	0	0	0	0	0	0	0	0	0
8	Sigma DP1S	0	0	0	0	0	0	0	2	0	0	0	0	1	0	29	0
9	Sweex WC001 100k USB	0	0	0	3	0	6	0	0	7	0	0	0	0	0	14	23.33
10	Vodafone 710	1	0	0	6	0	0	0	23	0	0	0	0	0	0	0	0
11	Blackberry Curve 9360	0	0	0	0	0	0	0	0	0	0	0	0	0	0	30	0
12	Logitech QuickCam Communicate STX	0	0	0	10	1	0	0	19	0	0	0	0	0	0	0	0
13	Samsung Galaxy S3 mini	0	0	0	0	0	0	0	0	0	0	0	0	0	0	30	0
14	Trust WB-1400T	0	0	0	0	0	0	5	0	0	0	0	0	0	25	0	83.33
15	Sony Cyber-shot DSC-S800	0	0	0	0	0	0	0	0	0	0	0	0	0	0	30	100.00

**Table 6.** Comparison of camera identification of Experiment 2 and Experiment 3(%).

Camera	Per-class accuracy Experiment 2	Per-class accuracy Experiment 3
Top-1 accuracy	21.57	31.11
Top-5 accuracy	69.61	74.00
Blackberry Curve 9300	0	0
Creative Live! Cam Video IM	56.67	96.67
Canon Ixus 220HS	0	3.33
Motorola V360	3.33	40.00
Panasonic Lumix SZ5	0	50.00
Philips SPC 200NC	100.00	100.00
Canon Powershot SX210	0	0
Sigma DP1S	6.67	0
Sweex WC001 100k USB	0	23.33
Vodafone 710	0	0
Blackberry Curve 9360	0	0
Logitech QuickCam Communicate STX	0	0
Samsung Galaxy S3 mini	0	0
Trust WB-1400T	100.00	83.33
Sony Cyber-shot DSC-S800	0	100.00

### Additional experiments

Other than the main experiments, there were additional ones that were performed to improve the results. It is important to mention that one of the databases consisted of six different camera models. In the database, there were 4–10 different cameras of the same model. During all the training procedures, the results were unsatisfactory. The top accuracy was 34%. Furthermore, the confusion matrix showed that most of the images were not identified correctly and even the Top-5 accuracy was at 23% showing that the questioned image was not even reached the Top-5 predictions. As a result, it was concluded that the individualization of a camera was unsuccessful with the current CNN. The network may need additional layers or other modifications.

## Discussion and conclusion

By modifying the AlexNet, the training of the model was improved, which was observed from the high accuracy rate (80%–90%) of the Experiments 1–3. During Experiment 1, DIGITS showed to have correctly identified six out of 10 cameras with a success rate higher than 75%. Nonetheless, during Experiment 2, it was observed that when the number of classes increased to 17, the success rate of the model decreased dramatically as only three cameras were correctly identified. From the results of this experiment “Samsung ST30” and “Nikon Coolpix L27” seemed to interfere with the outcome, since other cameras were falsely identified as one of those two. This indicated that the results of Experiment 1 were not as promising as initially anticipated.

A possible explanation for this is that the PRNU signal is based on the quality of the sensor and the artefacts introduced during the production process of the camera. Some manufacturers may use the same or similar imaging sensors, which could result in similar PRNU noise patterns. In addition, low quality sensors might generate more artefacts than high quality sensors. As it is mentioned in the article by Gloe et al. [14], the authors experienced unexpected difficulties because of the presence of diagonal artefacts in one of their samples. Furthermore, the exposure time of images was also another factor that could influence camera identification with the use of PRNU noise patterns. Nevertheless, most of their experiments confirmed that a PRNU camera identification has a very high reliability [14]. The afore-mentioned study conflicts with the theory that PRNU noise pattern is stochastic in nature and thus advantageous for achieving individualization by Fridrich [2], as there are cameras with non-unique artefacts visible in their PRNU noise patterns. Artefacts are inconsistencies and “flaws” which are created during the production of a specific camera. Cheap cameras, which probably contain low quality sensors, include many artefacts. These are visible in the PRNU, making it easier for DIGITS to detect them and identify their origin. Some examples are “Creative Live! Cam Video IM”, “Philips SPC 200NC”, “Sweex WC001 100k USB” and “Trust WB-1400T” which are all web cameras and thence probably cheap with low-quality sensors. On the other hand, expensive cameras like “Nikon Coolpix L27” probably contain high-quality sensors which create only a few distinct artefacts, making them more distinguishable from other cameras. As a result, these two categories can be identified using DIGITS or another CNN. However, medium priced cameras with adequate quality sensor will be falsely identified.

In addition to the above-mentioned remark, in cameras containing low-quality sensors, the artefacts may be non-unique, but common among different cameras. Therefore, the detection of these artefacts might confuse a neural network and lead to false identification. As observed from the results of this study, cheap cameras which more likely contain low quality imaging sensors, like “Samsung ST30”, interfered with the identification of other cameras Experiment 2 [4]. This could explain why DIGITS could not distinguish the similarities and the differences between the different camera models. Furthermore, in an attempt to create a database containing more than one camera of the same model, the accuracy rate was extremely low providing further proof that individualization of a camera could not be achieved with neural networks.

It is important to mention the fact that in a similar research by Tuama et al. [3], and Baroffio et al. [10], the experiments were successful by using a different form of filtering the original image. The purpose of this research was to combine PRNU noise pattern and deep learning for camera identification. All the modifications to AlexNet were based on the article by Tuama et al. [3]. Since the purpose of this study is camera identification with the use of PRNU noise patterns with deep learning, the pre-processing of the images and the creation of the database were made under different conditions comparing with the paper by Tuama et al. [3]. The authors used a high pass filter to all the images based on the method by Qian et al. [15] and subdivided each image to fit the AlexNet conditions, while, for this study, the database was created exclusively by PRNU noise patterns. These noise patterns were extracted with the use of the NFI tool “PRNU Compare” which was based on the proposed method by Gisolf et al. [7,8].

Noteworthy is also the fact that during this research, cropped images have been used which was a pre-requisite of the AlexNet. This may have also affected the outcome of the study since data may have been lost during cropping. As a result, it would be beneficial in future studies to modify the AlexNet in order to accept as input, higher resolution images. Moreover, it is important for further research to create a much bigger database than the one used in this study, which contained only 200 images per class. A bigger database will feed the program with much more information which is essential for a deep learning technique to succeed. Finally, the use of a database consisting entirely of flat field images could prove to be an improvement over a database containing both flat field and natural images.

To conclude, it was observed that the database plays a very important role to the training procedure; not only its size but also its content. Using a database with more images for each class may improve the accuracy value of the trained model. Nevertheless, it cannot guarantee successful identification of a camera. This study showed that the tested CNNs cannot achieve individualization with PRNU patterns.
